# A step-by-step overview of the dynamic process of epitope selection by major histocompatibility complex class II for presentation to helper T cells

**DOI:** 10.12688/f1000research.7664.1

**Published:** 2016-06-09

**Authors:** Scheherazade Sadegh-Nasseri

**Affiliations:** 1 Department of Pathology, Johns Hopkins University School of Medicine, Baltimore, Maryland, 21205, USA

**Keywords:** Epitope Mapping, Antigen Processing Machinery, immunodominance

## Abstract

T cell antigen receptors (TCRs) expressed on cytotoxic or helper T cells can only see their specific target antigen as short sequences of peptides bound to the groove of proteins of major histocompatibility complex (MHC) class I, and class II respectively. In addition to the many steps, several participating proteins, and multiple cellular compartments involved in the processing of antigens, the MHC structure, with its dynamic and flexible groove, has perfectly evolved as the underlying instrument for epitope selection. In this review, I have taken a step-by-step, and rather historical, view to describe antigen processing and determinant selection, as we understand it today, all based on decades of intense research by hundreds of laboratories.

## Introduction

T cells and B cells are two major components of the adaptive and specific immune system. While B cells can recognize antigens as a whole via their B cell receptors, T cells can only see a processed form of antigens, that is, short peptide sequences bound to the proteins of major histocompatibility complex (MHC) class I and class II. There are also two major classes of T cells: cytotoxic T cells (Tc), which are restricted to MHC class I, express CD8 accessory molecules on their cell membranes, and function by killing their targets, and helper T cells, identified by restriction to MHC class II and expression of CD4 accessory molecules. Helper T cells function by producing cytokines that help B cells in antibody production and isotype switching, as well as helping CD8 ^+^ T cells to develop into memory cells. Helper T cells are divided into several subclasses, each having different functions ^1^. CD8 ^+^ T cells are generally responsive to antigens such as viruses which have been endogenously expressed, while helper T cells present antigens taken up from exogenous sources. The machinery that best generates short peptides that bind to MHC molecules is present in antigen-presenting cells (APC). While a variety of cells might be able to process antigens under certain circumstances, dendritic cells (DC), B cells, and macrophages are considered professional APCs. Antigen processing for presentation by MHC class I follows a different biosynthetic pathway than that of MHC class II ^2, 3^. In the following sections, I focus on MHC class II, discussing different aspects of epitope generation and selection as assisted by the accessory molecules and processing enzymes that allow flawless completion of this complex process. At the end, I will briefly review attempts at identifying peptides that bind MHC molecules.

## MHC molecules have optimal structures for presenting antigens

For the presentation of antigen to helper T cells, APC must achieve an ambitious goal. One or few epitopes from a given antigen must be selected to fit stably and specifically in the groove of MHC class II. However, the number of possible epitopes to bind each MHC molecule is infinite, while each individual carries a maximum of six to eight MHC class II alleles. How is it possible for those few MHC molecules to bind peptides stably but non-specifically? The crystal structure of MHC class II, HLA-DR1 ^4^, revealed two sets of interactions with the bound peptide: side chains of peptides interacting with five pockets (pockets 1, 4, 6, 7, and 9), and a series of 13 H-bonds that formed between peptide main chains and the non-polymorphic residues of the MHC groove. It appears that by adopting a combination of pockets that accommodate peptide side chains, the MHC molecule meets the specificity criterion, and by forming H-bonds, complex stability can be achieved.

The next challenging demand from the MHC class II structure is to ensure that peptides from the exogenous antigens bind to the groove of MHC II efficiently. The solution here is provided by evolving a peptide-binding groove that is highly flexible and susceptible to collapsing in the absence of a bound peptide ^5– 7^. I will write more about this topic later.

### Resistance to SDS-mediated denaturation as a means of detecting peptide binding *in vivo*

The flexibility of the groove is a theme that I shall revisit throughout this review. To appreciate this concept, the readers of this review are likely to benefit from a brief history of peptide binding to MHC class II as part of its folding. Harden McConnell’s group was the first to realize that there were kinetic and structural intermediates in peptide binding to MHC II ^8– 11^. Using a simple SDS-PAGE assay where samples were kept at room temperature, the team demonstrated that naturally formed peptide/MHC (pMHC) complexes, purified from APC, migrated differently if peptides were dissociated. A loosely bound pMHC, or a peptide free MHC molecule, migrated as a slower migrating species that was named floppy dimers, relative to a faster migrating species called compact dimers. Compact dimers were shown to contain peptide, and unstably bound pMHC dissociated into single chains in SDS-PAGE (SDS sensitive) ^10, 11^. Importantly, when peptides that could form stable complexes with MHC II molecules were added back to MHC II, the partially unfolded floppy dimers and the dissociated chains reverted to compact conformations ^6, 7^. It was of great significance that the *in vitro* findings were confirmed in cells. In pulse-chase experiments, analyzed by SDS-PAGE, newly synthesized MHC II molecules that were not in complex with peptides from exogenous sources (pulse) dissociated into single chains, whereas MHC class II molecules that had formed complexes with exogenous peptides (chase) migrated as SDS-stable dimers ^12, 13^. By this criterion, class II molecules were shown to associate with peptides in the endocytic route prior to cell surface expression, a process that requires proteolytic digestion of the protein antigens ^13^. It was also shown that SDS stability did not always correlate with the stability of pMHC complexes; altered MHC mutants bound peptides loosely yet formed the characteristic SDS-stable conformation ^14^. Those original observations have been confirmed through numerous techniques over two decades of research by independent laboratories ^15– 19^.

The remarkable characteristic of MHC class II to resist SDS denaturation when in complex antigenic peptides allowed new discoveries that revealed steps in MHC class II synthesis, association with invariant chain (Ii), exposure to antigen-processing enzymes, MHC II trafficking, interaction with accessory molecules, peptide binding and editing, and more, as discussed below.

## Antigen-processing machinery

Antigen presentation to CD4 ^+^ T cells begins by the uptake of exogenous antigens by APC and their processing by proteolytic enzymes, mainly different cathepsins (Cat). The process involves transfer through a series of vesicular subcompartments containing suitable denaturing environments, a variety of accessory molecules and molecular chaperones, as well as cathepsins ^20^. Cathepsins present in processing compartments contribute by cutting and trimming of the protein antigens.

### Cathepsins

Antigen-processing proteases, or cathepsins, are amongst the most significant contributors to antigen processing and act as exoproteases, or endopeptidases ^21^. Expression levels and the activity of cathepsins are highly regulated in different cell types and activation states. Historically, two main roles have been described for cathepsins in antigen processing: to cleave off Ii and to process protein antigens. A new important function for cathepsins in the selection of immunodominant epitopes has recently been described and will be discussed later ^22^. Some of the most extensively studied cathepsins are CatB, CatD, CatL, and CatS ^23– 27^. CatS was reported to be involved in Ii cleavage and antigen processing ^28– 31^. Recent studies by Kim *et al.* ^22^ using a cell free processing system showed that inclusion of only three cathepsins (CatB, CatH, and CatS) was sufficient to mimic the processing conditions necessary to produce the immunodominant epitopes from several protein antigens. It is of note that cathepsins involved in antigen processing require acidic pH for their proteolytic function, which itself is highly regulated. Indeed, DC maturation promotes activation of vacuolar proton pumps and enhances lysosomal acidification ^32^.

### Invariant chain

Upon synthesis, every allele of the MHC II heterodimers forms complexes with a third nonpolymorphic chain, called class II Ii, which acts as a chaperone in folding among its several other functions. The Ii was first discovered by Jones and McDevitt, and was found to bind to all MHC II alleles ^33^. After intense research by numerous laboratories, it became clear that Ii acts as a chaperone for the newly synthesized MHC II ^34^. Its structure is rather segmented, each having a different function. Using nuclear magnetic resonance (NMR) techniques, Jasanoff *et al.* ^35– 37^ reported that a soluble recombinant Ii in complex with MHC II was mainly disordered except for two regions, one that included a region of 24 amino acids corresponding to the class II-associated Ii peptide (CLIP) and the other which participated in trimerization of the Ii to form nonomeric assemblies. The CLIP region binds in the peptide-binding groove of class II molecules in the endoplasmic reticulum (ER) and remains bound in cleaved form in the peptide-loading compartment, where the rest of the Ii is cleaved off by cathepsins ^38– 41^. Another important function of Ii is to target the newly synthesized MHC class II to the proper endocytic compartments ^42^, where it intercepts with protein antigens. The specialized endosomal compartments, called MIIC or CIIV ^43– 47^, were discovered as lysosome-like compartments which contained all necessary machinery for the processing of antigen and the optimal binding and selection of the peptides for presentation to the T cells ^48^. These vesicles are dense membranous structures that fall between the early endosomes and lysosomes in density, as well as their denaturing environment to include acidic pH ^49^, denaturing and proteolytic enzymes.

In addition to its other chaperoning functions, binding of Ii to MHC II was originally considered a means of preventing unproductive binding of peptides present in the ER ^39^. However, when the first Ii knockout mice were reported, it turned out that the MHC II molecules of Ii-deficient mice did not bind as many peptides as did the MHC II molecules of Ii-sufficient mice ^50, 51^. With better understanding of the flexibility and instability of the peptide-binding groove in the absence of a bound peptide ^52– 56^, it became clear that an unappreciated function of the CLIP region is that it acts as a surrogate peptide for shaping the MHC II groove. These studies demonstrated that the MHC class II groove collapses in the absence of a bound peptide and that a poor binding peptide, such as CLIP, maintains the groove in proper conformation. Upon dissociation of CLIP, a peptide-receptive conformation is generated that can scan peptides or unfolded proteins in the antigen-processing compartments ^55, 56^. Ii shuttles the MHC II molecules to MIIC, where Ii is proteolyzed by different cathepsins, including CatS, until only the CLIP fragment remains bound in the MHC II peptide-binding groove ^26, 31, 57, 58^. CLIP must then be exchanged for exogenous peptide, a function best performed by the accessory molecule HLA-DM in humans or H2-M in mice (DM, from now on) ^59^.

### HLA-DM

DM is a non-polymorphic MHC II-like molecule that does not bind peptides itself ^60^ but is necessary for the efficient displacement of CLIP from the MHC groove ^59, 61– 69^. The significance of DM in antigen presentation was first discovered through the observation that some APC lines did not process protein antigens for presentation to specific T cells. Those cell lines were found to have defective DM genes ^70^. Later, it was discovered that HLA-DR molecules from an antigen-processing mutant cell line were occupied with invariant chain peptides ^61, 71^. Hence DM was critical for the removal of CLIP and its exchange for the exogenous peptides.

***Mechanism of DM function.*** Understanding the mechanism of the function of DM posed a problem for a long period of time, as it was generally believed that DM dissociated all bound peptides from MHC II molecules. This concept created a dilemma: how could any peptide remain bound in the groove of MHC II when every peptide was susceptible to dissociation? The problem was partially solved by the finding that not all peptides were equally susceptible to DM-mediated dissociation ^72, 73^. Certain peptides that would fit the MHC II groove and formed a rather rigid or compact conformation remained resistant to DM-mediated dissociation ^74– 84^. It was proposed that DM functions by recognizing conformations of pMHC II complexes that vary based on the nature of the bound peptides. For the best-studied MHC II molecule, HLA-DR1, it is well established that P1 interaction is the key determinant of pMHC II complex stability ^14, 52, 85^ and that peptides interacting non-optimally in the P1 pocket are highly susceptible to DM-mediated peptide exchange ^15, 74, 75, 80, 82, 86, 87^. DM interaction induces major conformational alterations in the P1 area of the MHC II groove, leading to destabilization of the bound peptide and preventing the formation of H-bonds, hence peptide dissociation. When peptide is released, a peptide-receptive MHC II is generated ^74, 86^, which can quickly sample a large pool of sequences from the available proteins.

**Figure 1.**
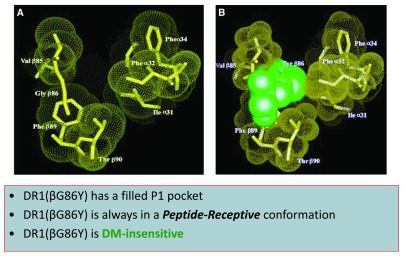
DR1(βG86Y) is always in a peptide-receptive conformation. Substitution of glycine for tyrosine at position 86 of DR1 beta chain generates a P1 pocket that is partially filled and resistant to DM-induced effects ^14^.

The significance of P1 in interaction with DR1 was demonstrated by a mutagenized DR1 that expressed a partially filled P1 pocket and failed to interact with DM ^15, 74, 82^. The mutant molecule, DR1(bG81Y), carrying a single amino acid change from G to Y, was constitutively peptide receptive and migrated as compact dimers in gentle SDS-PAGE ( Figure 1). Indeed, the DR1(bG81Y) molecule resembled murine I-E ^k^, which has a shallow pocket 1 ^15^ and itself is resistant to the DM editing function. In agreement with the structural characteristics of I-E ^k^, DM knockout haplotype K mice did not show the characteristic defects in peptide binding and occupancy with CLIP associated with H-2 ^b^ mice ^88^. Thus, DM can only affect peptide exchange in MHC II alleles of certain structural requirements ^89^.

With the solution of the crystal structure of the DM/DR complex using a cleverly designed DR1/peptide complex that allowed for the DR1 groove to remain open, it was established that DM would bind the P1 pocket of HLA-DR molecules if empty and would remain bound until a P1 filling peptide bound the groove ^17, 84, 90^. The above findings were complemented by the measured thermodynamics of peptide binding to DR1, indicating that a greater entropic penalty, versus a smaller penalty, was associated with structural rigidity rather than with the flexibility of the pMHC complexes ^87^. Consistent with the previous reports, the authors found that DM senses flexible complexes, in which the P1 area residues are rearranged at a higher frequency than in more rigid complexes. Moreover, a new and unexpected observation reported that conformational changes in the P1 area could be negated if the P9 pocket anchor residue of peptide was substituted for a stronger binding residue ^16^. The findings suggest that an overall dynamic MHC II conformation, in addition to P1 pocket occupancy, determines susceptibility to HLA-DM-mediated peptide exchange and provides a molecular mechanism for HLA-DM to efficiently target poorly fitting pMHC II complexes, editing them for more stable ones. Hence, in addition to the removal of CLIP, DM helps in shaping epitope selection (more details to follow).

***Biological significance of DM.*** As discussed earlier, DM plays an important role in selecting the right peptides that can stay in the groove of MHC II long enough for T cell recognition ^91^. This characteristic of DM contributes to T cell immunity in a significant way. Lymphocytes usually respond to a small proportion of the potential determinants on a protein antigen defined as “immunodominant” ^92^. Immunodominant epitopes are the essential targets of the immune response against infectious diseases, cancer, autoimmune diseases, and allergy. Consequently, much attention has been devoted to the understanding of epitope selection and immunodominance. However, in spite of the complexities of antigen processing and presentation, T cell epitope discovery has been a challenging task. Some of the factors contributing to immunodominance are described below.

***Epitope accessibility and its relation to immunodominance.*** Among many contributing factors to an epitope gaining immunodominance is how accessible the location of sequence is to the groove of the MHC II molecule and/or to the processing enzymes ^93^. The denaturing environment in the antigen processing compartments (acidic pH and reducing conditions) helps to partially unfold protein antigens to reveal hidden epitopes. Of particular interest is a specialized enzyme, gamma-interferon-inducible lysosomal thiol reductase (GILT), that releases disulfide bonds in proteins ^94^, making denaturation more efficient. In support of the significance of GILT in the release of dominant epitopes is the fact that GILT-deficient mice failed to present buried determinants of hen egg lysozyme (HEL) and an HA protein of influenza; HEL and HA both have four disulfide bonds ^95, 96^. Support of the “epitope accessibility” model for immunodominance comes from accumulated evidence that many of the naturally selected epitopes localize on flexible strands of protein antigens ^93^ or at the C- or N-terminus of protein antigens ^97– 99^. For a more comprehensive review on the subject of accessibility, the readers are referred to 102.

One question that might come to the mind of readers is how is it that the MHC II and their accessory molecules are not denatured in such an aggressive environment? It is of note that acidification of the antigen processing compartments in DC is developmentally regulated. Hence, the vacuolar proton pump that acidifies MIIC and activates cathepsins for processing of internalized antigens is activated only upon DC maturation ^32^. Also of importance is that MHC II and DM molecules resist denaturation and cleavage ^100^ by the harsh acidic pH and proteolytic conditions likely present in the late endosomes.

**Figure 2.**
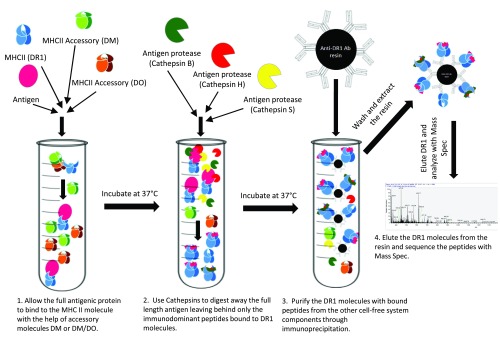
A reductionist cell free antigen processing system. Purified MHC class II and accessory molecules are exposed to full-length antigens and cathepsins under denaturing conditions. MHC class II molecules are then isolated and subjected to peptide elution and mass spectrometry ^100^.

***A cell free reductionist antigen processing system.*** A need for epitope accessibility together with the open-ended groove of MHC class II hint at binding of MHC II to the whole antigen rather than precut peptides. While there have been several examples of MHC II binding to full length antigens ^101– 103^, the prevailing dogma assumes that peptides are cut first, and then binding to MHC II and selection by DM takes place ^104^. However, direct evidence in support of binding of full length protein to MHC II and determinant selection by DM was put forth by the design and use of a reductionist cell free antigen processing system, which documented that full-length proteins, or a mixture of protein fragments, could be processed and the immunodominant epitopes could be selected by a minimal number of ingredients ^100^ ( Figure 2). The components of this minimalist system include MHC class II, HLA-DR, full-length denatured protein antigen, three processing enzymes, cathepsins S (an endopeptidase), B, and H (exopeptidases), and HLA-DM, all placed in a tube in acidic pH. After allowing time for processing of the antigen, peptide binding, and DM editing, DR molecules, now bound to the selected epitopes, are immunoprecipitated and the bound peptides are released by exposure to low pH and are then subjected to mass spectrometry. In the following steps, the identified peptides, which usually are not very many, will be tested for immunogenicity in HLA-DR1-expressing Tg mice immunized with the full-length protein antigens. The results were quite pleasing: peptides identified by the reductionist system were immunodominant epitopes because they recalled nearly full T cell responses. Importantly, even when tested in human volunteers, the peptides identified by the reductionist system proved to accurately reflect antigen processing in human APCs ^100^. It is significant that the immunodominant epitopes were identified when DM was included in the system whereas, in the absence of DM, other non-dominant epitopes were also found among the eluted peptides.

The results from the reductionist system suggested that DM plays a key role in the selection of the immunodominant epitopes from exogenous antigens ^22, 100, 105, 106^. In a later extensive study, Yin *et al.* compared affinity, intrinsic dissociation half-life, and DM-mediated dissociation half-life as well as two epitope prediction algorithms (more below) for many peptides derived from the entire Vaccinia genome for inducing CD4 ^+^ T cell responses. The results confirmed that pMHC II complex kinetic stability in the presence of DM was the determining factor for distinguishing the immunodominant epitopes from the non-dominant bound peptides ^107^. In agreement with results from the reductionist system, these analyses demonstrated that DM editing governs peptide immunogenicity by favoring the presentation of peptides with greater kinetic stability. However, it is of note that not all stable pMHC complexes are immunodominant. Moreover, autoimmune epitopes may or may not be resistant to DM-mediated dissociation ^22^.

**Figure 3.**
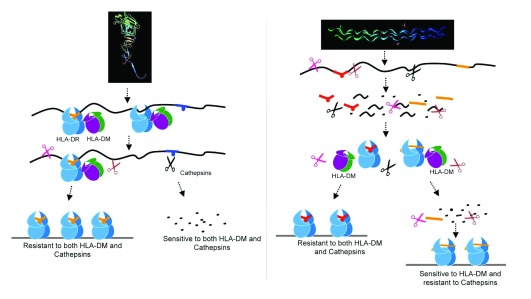
Auto-antigens and pathogen-derived antigens are processed differently. Influenza-derived HA protein (left) is captured as full-length denatured protein or large fragments by MHC class II, edited by DM, and then is exposed to cathepsin digestion. An example of an auto-antigen, collagen (right), is first cut into short peptides and then binds MHC class II; it may be either sensitive or resistant to DM-mediated editing ^22^.

The use of the cell free reductionist system also enabled the authors to gain a new understanding of dominant epitope selection ^22, 105, 106, 108^. The authors showed that peptides derived from pathogens, or autoantigens, behaved differently in response to DM. For autoantigens, resistance to DM-mediated dissociation was not a required criterion, whereas for pathogen-derived dominant epitopes, DM resistance was a crucial factor. Immunodominance emerged as a result of the combined effects of DM and the antigen processing cathepsins. Autoantigen-derived immunodominant epitopes were resistant to digestion by cathepsins in the system, whereas pathogen-derived epitopes were sensitive. As such, sensitivity to cathepsins necessitated the capture of pathogen-derived epitopes by MHC II prior to cathepsins processing, and resistance to DM-mediated-dissociation preserved those epitopes from pMHC release and degradation ^22^. The overall findings demonstrated that immunodominance is established by the higher relative abundance of the selected epitopes that survive cathepsins digestion either by binding to MHC II and resisting DM-mediated-dissociation or by being chemically resistant to cathepsin degradation. Non-dominant epitopes were found to be sensitive to both DM and cathepsins ^22^ ( Figure 3). Consistent with the autoimmune epitopes being resistant to proteolysis is the finding that large numbers of peptides derived from autoantigens have been identified in normal pre-nodal afferent human lymph ^109, 110^. The lymph peptidome must have resisted the variety of catabolic enzymes present in tissues, the function of which remains to be understood.

***Possible role of DM in the quality of the peptide/MHC II complex.*** Intriguingly, there are reports documenting that some autoimmune T cells might discriminate among peptides that form complexes with MHC II in the presence or absence of DM. A clear example has been pioneered by Unanue and colleagues, who showed two types of T cells: type A that recognize pMHC generated by intracellular processing machinery including DM and type B T cells that recognize pMHC formed in the absence of DM ^111, 112^. Of outstanding interest is that autoreactive CD4 ^+^ T cells specific for an insulin peptide were type B T cells; they did not recognize the insulin protein when processed by APC and, as such, could not have been deleted during thymic education ^113^. These findings suggest that the topology of the complexes formed in the presence or absence of DM might be different. The findings of the Unanue and Sadegh-Nasseri laboratories hint at the possibility of a different path for antigen processing for autoimmune epitopes. As discussed, autoimmune epitopes may or may not be sensitive to DM-mediated dissociation, and they are highly resistant to the proteases in antigen processing ^22^. Hence, it is very likely that for some autoimmune diseases, autoantigens are generated in an extracellular matrix where many proteases are already at work. The core epitopes that survive such a protease-rich milieu may get a chance to bind to the empty MHC II molecules expressed on APC membranes or in the early endosomal compartment where DM is not active ^114^. Such complexes would not be edited by DM and would fit the required ligand characteristic for type B T cells. Alternatively, some of the larger antigen fragments might be processed in the early endosomes where DM does not contribute to peptide editing, leading to the generation of type B pMHC complexes.

### HLA-DO

In addition to DM, another non-classical MHC class II accessory molecule, HLA-DO, H2-O in mice, DO from now on, is known to play a role in peptide exchange ^115– 117^. Of importance, DO has restricted tissue expression; it is mainly expressed in B cells and thymic medullary epithelium, where thymic deletion takes place. In addition, certain subsets of DCs express DO under different conditions. Cellular trafficking of DO depends on DM. Understanding how DO contributes to antigen processing has been a challenge for decades. Two recently solved crystal structures, DM/DO and DM/DR1, suggested that the DM/DO interface is shared with the DM interface with DR1 ^90, 118^. These findings were interpreted to imply that DO might act as a competitive inhibitor of DM in interaction with DR. While this model has previously been advocated ^117^, peptide binding association and dissociation kinetics conducted in the presence of DM, and/or DM/DO, put forward an alternative mechanism ^115, 116^. It was shown that DO binds to DR molecules. Rather than inhibiting DM, it was demonstrated that DO works together with DM to increase the binding of peptides that formed DM-resistant complexes with DR, while reducing the binding of peptides that are DM sensitive. Furthermore, the positive and negative effects of DO on peptide binding were shown to be restricted to the association phase, as the peptide dissociation phase remained unaffected by DO. Interestingly, DO could only bind to a peptide-receptive rather than peptide-occupied DR1. Because DO is always in complex with DM, and DM works by generating a peptide-receptive conformation, the authors proposed a model to suggest that DM might dissociate pMHC, leading to a peptide-receptive DR that can be stabilized by DO. Thus, DO and DM work in synergy for optimizing peptide exchange and for selecting the DM-resistant peptides. The combined efforts of all the molecules discussed above, and perhaps others whose identities are yet to be discovered, lead to an impeccable selection process for the immunodominant epitopes for MHC II groove occupancy and transport to the APC external membrane for CD4 ^+^ T cell stimulation.

## Search for the immunodominant epitopes

Clearly, finding peptide epitopes that bind to MHC molecules and represent a given antigen is highly desirable for use in therapeutics and vaccine designs. A variety of methods have been used for this purpose. Among those is the screening of hundreds of overlapping synthetic peptides that span the entire sequence of an antigen of interest for binding to MHC II molecules. The screening may involve biophysical methods to measure MHC II binding, T cell readout assays, or tetramer-guided epitope mapping. Hundreds of tetramers would be made using hundreds of overlapping peptides for detection of the T cells reactive to the antigen ^119^. These methods are generally labor intensive, costly, and often insufficient because, with the exception of tetramer-guided mapping, they do not take into account steps involved in the processing of antigens as it happens in the cellular environment. In the identification of autoimmune epitopes, the task is even more arduous because autoimmune epitopes, in addition to the characteristics discussed above, often include post-translational modifications ^119^, which makes screening of the peptides based on the amino acid sequences of the proteins rather hopeless.

### Computational approaches and mass spectrometry

Computational approaches are popular alternative methods for predicting possible epitopes that bind MHC class II molecules with high affinity. The guiding principle in all computational methods is the structural information available on different MHC grooves. Although somewhat successful for predicting MHC class I epitopes, computer prediction algorithms have been generally unsuccessful in identifying CD4 ^+^ T cell epitopes ^120– 122^. The open-ended MHC class II groove versus the better-defined pocket-fitting residues for MHC class I adds to the complexity of structure-based predictions exponentially. In addition, there is no computational way yet available for predicting how DM and DO would contribute to the epitope selection ^107^.

The next popular method in epitope discovery is the use of mass spectrometry. Mass spectrometry for the identification of peptides eluted from MHC class I and class II was first reported in the early 1990s ^123^ and required large amounts of purified MHC molecules. Thousands of self-peptides are generally eluted from MHC molecules isolated from even uninfected APCs. In recent years, because of the great progress in the development of highly sensitive instruments for mass spectrometry, the need for high quantities of starting numbers of APCs (~10 ^11^) has been significantly reduced ^124^. However, for an accurate determination of the dominant epitopes by peptide elution, it is necessary to utilize quantitative mass spectrometry because immunodominant epitopes are often displayed at the highest prevalence ^22^, yet quantitative mass spectrometry has its own associated extreme complexities ^125^.

## Concluding remarks

As discussed above, antigen processing is a complex multistep process that has evolved for the identification of the best-fitting epitopes for T cell recognition and functions. A number of chaperones together with the uniquely evolved MHC class II molecular structure, which requires a peptide as part of its fully folded state, contribute to this ultimate goal. While much has been learned over the past decades about antigen processing and presentation, because of the complexities involved, a successful peptide prediction strategy has yet to be discovered. The divergent paths for the processing of proteins of exogenous versus self-antigens open up new fields to explore. Understanding the biology of DO and its effects on the development of autoimmune diseases is another area that has remained challenging. Finally, the expression of MHC II upon T cell activation and its impact on immune responses begs further research. We can only hope that current and future research will focus on these unanswered questions.
